# The need for early intervention for psychosis to persist throughout the COVID-19 pandemic and beyond

**DOI:** 10.1017/ipm.2020.56

**Published:** 2020-05-21

**Authors:** B. O’Donoghue, K. O’Connor, A. Thompson, P. McGorry

**Affiliations:** 1 Orygen, 35 Poplar rd, Parkville, VIC 3052, Australia; 2 Centre for Youth Mental Health, University of Melbourne, Melbourne, Australia; 3 Home Based Treatment Team and Responsive Early Intervention for Psychosis Service (RISE), South Lee Mental Health Services, Co Cork, Ireland; 4 National Clinical Lead Early Intervention for Psychosis, Health Service Executive, Dublin, Ireland

## Abstract

In the last three decades, early intervention for psychosis (EIP) services have been established worldwide and have resulted in superior symptomatic and functional outcomes for people affected by psychotic disorders. These improved outcomes are a result of reducing delays to treatment and the provision of specialised, holistic interventions. The COVID-19 pandemic poses significant challenges to the delivery of these services, such as undetected cases or long delays to treatment. Furthermore, the COVID-19 pandemic will likely increase the mental health needs of communities, including the incidence of psychotic disorders. In this perspective piece, we provide suggestions as to how EIP services can adapt within this environment, such as utilising novel technologies. Finally, we argue that despite the economic consequences of the pandemic, the funding for mental health services, including EI services, should be increased in line with the need for these services during and beyond the pandemic.

The last three decades have seen the advent and worldwide adaptation of early intervention for psychosis (EIP) services (Jackson & McGorry, 2009), which result in superior symptomatic and functional outcomes (Correll *et al*. 2018). The rationale behind these services is that by reducing delays to specialised treatment [referred to as the duration of untreated psychosis (DUP)], this contributes strongly towards better outcomes (Marshall *et al*. 2005). The DUP consists of two components. The first delay can occur between the onset of symptoms and the time by which an individual or their caregivers seeks help, referred to as the ‘help-seeking delay’. The second delay can occur from the onset of help-seeking by the individual or their caregiver to the time that they get the appropriate treatment for a psychotic disorder, referred to the ‘treatment delay’. Both delays can be considerable, with individuals often having multiple contacts with healthcare professionals before it is recognised that they may be experiencing a psychotic disorder (O’Callaghan *et al*. 2010). In the UK, it was found that there were considerable treatment delays, even for individuals already attending a mental health service, where it was not identified that they were suffering with a psychotic disorder (Birchwood *et al*. 2013).

Therefore, a core component of EIP services is reducing these delays, thereby reducing the DUP and improving outcomes. This task is considerable and requires large-scale, multifaceted interventions. Public education campaigns are required to inform people of the signs of psychosis (Krstev *et al*. 2004), particularly those who are likely to come into contact with young people, such as teachers, counsellors, social welfare officers and the police (Sutton *et al*. 2018). Young people with a first episode of psychosis (FEP) are often first seen by general practitioners, emergency department services or emergency services staff and interventions need to be targeted to these professionals to assist them in identifying psychotic symptoms and making the appropriate referrals (O’Callaghan *et al*. 2010). Furthermore, EI services need to respond rapidly and have a low threshold for conducting an assessment of someone suspected of having a FEP, even if that means conducting multiple assessments to identify one case of psychotic disorder (O’Donoghue *et al*. 2012). Employing the above strategies, EI services have been able to significantly reduce delays to treatments, for example, the Early Psychosis Prevention and Intervention Centre (EPPIC) in Melbourne, Australia has achieved a median DUPs of 8 weeks (Schimmelmann *et al*. 2008), compared to over 1 year in other countries where EI had not yet been introduced (Addington *et al*. 2015). This work to reduce delays is considerable, but it also must be ongoing, as cessation of these strategies leads to the return of long delays, as demonstrated by the TIPS (Scandinavian early Treatment and Intervention in Psychosis Study) study in Scandinavia (Joa *et al*. 2008).

At the turn of this decade, the COVID-19 pandemic has had an immediate and profound impact on our way of life and represents a healthcare crisis across the world. Social distancing, the most effective public health method for slowing the spread of the virus, has resulting in the closure of schools and universities, non-essential businesses, sports and recreational centres and places of religious worship (Prem *et al*. 2020). Social gatherings are also limited, and in some jurisdictions, people are only permitted to be in close proximity to their immediate family. While the above measures are clearly necessary to manage the pandemic and reduce the associated mortality, they will have a profound effect upon how we identify and provide treatment for people with major mental illnesses including those experiencing a FEP. Late adolescence and early adulthood are the peak periods for the onset of psychotic disorders (McGorry *et al*. 2011), but with the closure of the places where young people are likely to attend, the original targets for educational campaigns are no longer viable. Anecdotally, we are hearing from medical colleagues that people are now more hesitant about attending general practitioners and emergency departments for non-urgent matters, for fear of coming in contact with people infected with COVID-19. This essentially means that the main strategies for reducing treatment delays for people experiencing a FEP have suddenly become defunct. To further complicate things, identifying the subtle signs of early psychosis can be challenging enough, but in a world that has suddenly been consumed with anxiety, fear, social withdrawal and even widespread ‘conspiracy theories’ (Ahmed *et al*. 2020; Li *et al*. 2020), the task becomes even harder.

This could mean that following the pandemic, if steps are not taken to identify cases of FEP, there could be an increase in the number of people with a very long DUP. This is similar to the situation that EI services experience when they are established, as they can identify previously undetected case with a very long DUP (O’Donoghue *et al*. 2014). If the strategies to reduce delays cannot be employed and access to referral and treatment is hindered, then it is likely that the DUP will be prolonged for those experiencing a FEP throughout the pandemic. Following this, there would be a large ‘backlog’ of individuals with a very long DUP, who would present with a more enduring disorder and thereby less likely to make a full and sustained recovery (Santesteban-Echarri *et al*. 2017). Furthermore, there would be the regular, expected number of new cases of FEP, and EI services would struggle to provide the comprehensive treatments required for all of these new cases simultaneously.

When cases are identified during the pandemic, there will be further challenges in providing full, holistic assessments and treatments. The other complimentary explanation for improved outcomes with EI services is that they provide comprehensive, specialised treatments for those with a FEP within a critical period. Alongside antipsychotic medication, there are a range of psychological, vocational and family interventions that need to be delivered in order to achieve a full and sustained recovery (Killackey, 2009). The physical health of the affected individual also needs to be managed, in addition to any concurrent problems, such as substance abuse. Providing these multi-faceted interventions in an environment of social distancing and reduced in-person appointments poses a further challenge.

Therefore, throughout the COVID-19 pandemic, EIP services must be sustained or their roll out continued.

What can EI services do to adapt to the current environment? First, they need to monitor referral rates to their services in real time and compare them to the same period of the previous year, as any variation in the incidence of psychotic disorders in the same area over time is likely to be small (Kirkbride *et al*. 2009). A number of factors can precipitate the onset of a psychotic disorder, such as substance use or stress (Burns, 2013; Shah & Malla, 2015), and it is possible that the exposure to these factors during the pandemic may change. For some, the exposure to these factors may increase, such as financial stress for those who are unemployed, or their caregiver commitments may increase. While for others, stress may decrease with the cessation of school or substance use decline with the decrease in social gatherings. However, any major reduction in referrals is likely to reflect a decline in detection of cases and presentations to service, especially as the limited evidence to date suggests that the incidence of psychotic disorders is likely to increase during pandemics (Brown *et al*. 2020). If a service observed a decrease in referrals, they would need to ramp up their public education campaigns, albeit with different methods. Social distancing has resulted in people spending much more time in their homes, leading to an increase in activities such as watching television or streaming services and online activities (Dixit *et al*. 2020). It has already been established that television can be an effective method to increase the knowledge and awareness of the symptoms of psychosis in the general public and how to access care (Turner *et al*. 2014). Digital media advertisements are also a cost-effective and acceptable method by which to direct people with suspected psychotic symptoms to their local EI service (Birnbaum *et al*. 2017b). Therefore, it makes sense during the pandemic that educational campaigns are directed through these mediums in order to have the maximum impact. While these suggestions are being made in relation to psychotic disorders, the mental health needs of the community are likely to increase during and following the pandemic and the above approaches can be part of a broader strategy to improve and maintain access to care to mental health services.

Second, EI services should remove any existing barriers, such as the need for referrals from professionals. Direct referrals from affected individuals or their caregivers should be encouraged and facilitated. As already stated, in order to detect cases of FEP early, the EI service must be willing to conduct multiple assessments for each case. In Ireland, after the successful implementation of an educational campaign, it was found that for every three referrals for potential FEP, one was a case of FEP (O’Donoghue *et al*. 2012). If direct referral leads to an unmanageable amount of assessments, then services could consider implementing a screening instrument to triage referrals, although it needs to be considered that cases can be missed with this approach (O’Donoghue *et al*. 2018).

Third, EI services should be flexible in how they conduct assessments and provide treatment, such as via telehealth if clinically appropriate. This approach has been used successful and effectively for rural and remote areas already (Hensel *et al*. 2019) and has recently been adopted at the EPPIC service in Melbourne and other services worldwide. An evaluation of this new method of service delivery is ongoing, but anecdotal evidence indicates that it is well received by young people (who conduct a lot of their social interactions via this method anyway), and clinicians have reported that attendance rates have increased, due to the convenience and flexibility that it provides. However, it has been identified that training for clinicians must be a priority to ensure that these services are used effectively (Torous & Wykes, 2020). Also, considering that telehealth offers opportunities to reform mental health services, it needs to be ensured that the practice is maintained as a component of service delivery and not abandoned after the pandemic (Torous & Wykes, 2020). This is just one method of providing treatment and assessment remotely, and other options include the use of moderated online social therapy (Alvarez-Jimenez *et al*. 2013), which provide online psychological treatments that are moderated by a clinician, the use of mobile apps and real-time monitoring and virtual worlds/virtual reality. Importantly, both two of these methods, telehealth and moderated online therapy involve a clinician, as it has been demonstrated that online interventions are much more effective when delivered in conjunction with a clinician (Linardon *et al*. 2019).

However, there are some circumstances in which telehealth will not be feasible or appropriate, and for these individuals, in-person appointments will need to continue and assertive outreach conducted when clinically indicated. The monitoring of physical health and the management of any metabolic side-effects also need to provide if indicated. Services will need to be innovative in the manner by which they can manage this remotely. For example, it might consist of focusing on things that people can measure themselves at home, such as waist circumference, as this ‘vital sign’ is strongly correlated with cardiovascular disease (Ross *et al*. 2020). Lifestyle interventions should be encouraged, but overall people are less likely to be active with social distancing, and hence if there is indication of metabolic side-effects, then the corresponding pharmacological treatment such as statins or metformin should be considered early (Correll *et al*. 2013). EI services have also facilitated the development and evaluation of new treatments for early psychosis, and during the pandemic, a number of research centres have ceased recruitment (McDermott & Newman, 2020). It is not known how long social distancing will need to be in place, but new treatments are desperately needed in early psychosis and interventional research cannot be suspended indefinitely. Therefore, new ways of conducting clinical trials remotely in a safe and effective manner need to be established.

In the 1990s, the pioneering EI services for psychosis had to be creative in the approaches that they took to identify cases and configure services (McGorry *et al*. 1996). It is time again to harness this creativity and innovation. New methods for identifying those either at high risk for psychosis or experiencing a first episode need to be developed and there is promising work on this front. For example, it is possible to distinguish individuals with a diagnosis of schizophrenia by linguistics used on social media (Birnbaum *et al*. 2017a) and to identify those at risk of relapse (Birnbaum *et al*. 2019). Work is currently ongoing as to whether it is possible to identify those at risk of psychosis or experiencing a FEP using this method. EIP also includes specialised services for those who have been identified as being at ultra-high risk for psychosis (Yung & Nelson 2013), and these services typically provide care and monitoring for a defined period, most commonly 1 year. However, the risk of transitioning to a full-threshold psychotic disorder continues beyond this point, with 36% transitioning within 3 years (Fusar-Poli *et al*. 2012) and the risk is known to extend up to 10 years (Nelson *et al*. 2013). Therefore, during the pandemic, EI services could contact those discharged who were identified as ultra-high risk for psychosis and continue to provide monitoring remotely.

The advances made in EI have transformed mental health services and have resulted in improved outcomes for affected individuals and their families. In Ireland, EIP is one of the four National Clinical Programmes (NCP) and was developed in conjunction with the College of Psychiatrists of Ireland (Power, 2019). In 2019, the Health Service Executive funded three EIP demonstration sites in Ireland (Cork, South Lee, Meath & Sligo), bringing the number of EIP services in Ireland up to five (DETECT in Dublin & EIST, Cork North Lee). The full implementation of the EIP Model of Care was expected over the following 3 years and would result in National EIP service provision. It is critical that the implementation of the EIP Model of Care proceeds as planned. Now is the time to accelerate progress rather than reverse gains made.

Healthcare services, particularly Emergency Departments and Intensive Care Units, will need additional resources and manpower during the COVID-19 pandemic; however, this can be not be provided at the expense of mental health services. In addition to the current unmet need, the demands on mental health services are likely to increase during and after the pandemic and will likely be a major legacy of this public health crisis. The stakes are high as psychotic disorders and other mental health disorders are associated with high rates of suicide, more so in young people and in those left untreated (Robinson *et al*. 2011; Too *et al*. 2019). In addition to the healthcare crisis, there will likely be an economic recession in many countries worldwide, which will result in even less resources for underfunded health services. Mental health services can often be the victim in such circumstances. However, it needs to be highlighted that EI services are cost-effective and deliver considerable savings compared to standard treatment (Aceituno *et al*. 2019). Therefore, these services are an investment and should not be sacrificed for short-term savings. If mental health services involute to only providing acute psychiatric care, or worse still, staff are diverted out of mental health services, then the ability to undertake any preventative interventions will diminish and this will lead to increased morbidity in this patient population. In Australia, dynamic modelling of the adverse impacts on COVID-19 on unemployment, social distancing and mental health has indicated that suicides could increase by 25% for up to 5 years beyond the pandemic and that these deaths would overtake the number of deaths in the country directly attributable to the COVID-19 pandemic (Australian Medical Association, 2020).

As this pandemic continues, it will require innovation and imagination as to how we can continue to provide timely identification and treatment for individuals with early psychosis. There may be some potential benefits form harnessing technology to assess and treat individuals with early psychosis, but there are clearly major threats and challenges to this vulnerable group. Throughout the world, services will be attempting to transform themselves rapidly to address this need and there needs to be a mechanism by which services can inform each other as to what strategies are effective and which are not. The gains of the last three decades cannot be reversed but conversely must be built upon to meet the demands of the crisis and pivot to a 21st century model of care.

## References

[ref1] Aceituno D, Vera N, Prina AM, Mccrone P (2019). Cost-effectiveness of early intervention in psychosis: systematic review. British Journal of Psychiatry 215, 388–394. 30696495 10.1192/bjp.2018.298

[ref2] Addington J, Heinssen RK, Robinson DG, Schooler NR, Marcy P, Brunette MF, Correll CU, Estroff S, Mueser KT, Penn D, Robinson JA, Rosenheck RA, Azrin ST, Goldstein AB, Severe J, Kane JM (2015). Duration of untreated psychosis in community treatment settings in the United States. Psychiatric Services 66, 753–756. 25588418 10.1176/appi.ps.201400124

[ref3] Ahmed W, Vidal-Alaball J, Downing J, Lopez Segui F (2020). COVID-19 and the 5G conspiracy theory: social network analysis of Twitter data. Journal of Medical Internet Research 22, e19458. 32352383 10.2196/19458 PMC7205032

[ref4] Alvarez-Jimenez M, Bendall S, Lederman R, Wadley G, Chinnery G, Vargas S, Larkin M, Killackey E, McGorry PD, Gleeson JF (2013). On the HORYZON: moderated online social therapy for long-term recovery in first episode psychosis. Schizophrenia Research 143, 143–149. 23146146 10.1016/j.schres.2012.10.009

[ref5] Australian Medical Association (2020). Covid-10 impact likely to lead to increased rates of suicide and mental illness (https://ama.com.au/media/joint-statement-covid-19-impact-likely-lead-increased-rates-suicide-and-mental-illness). Accessed 7 May 2020.

[ref6] Birchwood M, Connor C, Lester H, Patterson P, Freemantle N, Marshall M, Fowler D, Lewis S, Jones P, Amos T, Everard L, Singh SP (2013). Reducing duration of untreated psychosis: care pathways to early intervention in psychosis services. British Journal of Psychiatry 203, 58–64. 23703317 10.1192/bjp.bp.112.125500

[ref7] Birnbaum ML, Ernala SK, Rizvi AF, Arenare E, Van Meter AR, De Choudhury M, Kane JM (2019). Detecting relapse in youth with psychotic disorders utilizing patient-generated and patient-contributed digital data from Facebook. Npj Schizophrenia 5, 17. 31591400 10.1038/s41537-019-0085-9 PMC6779748

[ref8] Birnbaum ML, Ernala SK, Rizvi AF, De Choudhury M, Kane JM (2017a). A collaborative approach to identifying social media markers of schizophrenia by employing machine learning and clinical appraisals. Journal of Medical Internet Research 19, e289. 28807891 10.2196/jmir.7956 PMC5575421

[ref9] Birnbaum ML, Garrett C, Baumel A, Scovel M, Rizvi AF, Muscat W, Kane JM (2017b). Using digital media advertising in early psychosis intervention. Psychiatric Services 68, 1144–1149. 28712355 10.1176/appi.ps.201600571

[ref10] Brown E, Gray R, Lo Monaco S, O’Donoghue B, Nelson B, Thompson A, Francey S, McGorry P (2020). The potential impact of Covid-19 on psychosis: a rapid review of contemporary epidemic and pandemic research. Schizophrenia Research. Published online ahead of print. doi:10.1016/j.schres.2020.05.005. PMC7200363 32389615

[ref11] Burns JK (2013). Pathways from cannabis to psychosis: a review of the evidence. Frontiers in Psychiatry 4, 128. 24133460 10.3389/fpsyt.2013.00128 PMC3796266

[ref12] Correll CU, Galling B, Pawar A, Krivko A, Bonetto C, Ruggeri M, Craig TJ, Nordentoft M, Srihari VH, Guloksuz S, Hui CLM, Chen EYH, Valencia M, Juarez F, Robinson DG, Schooler NR, Brunette MF, Mueser KT, Rosenheck RA, Marcy P, Addington J, Estroff SE, Robinson J, Penn D, Severe JB, Kane JM (2018). Comparison of early intervention services vs treatment as usual for early-phase psychosis: a systematic review, meta-analysis, and meta-regression. JAMA Psychiatry 75, 555–565. 29800949 10.1001/jamapsychiatry.2018.0623 PMC6137532

[ref13] Correll CU, Sikich L, Reeves G, Riddle M (2013). Metformin for antipsychotic-related weight gain and metabolic abnormalities: when, for whom, and for how long? American Journal of Psychiatry 170, 947–952. 24030606 10.1176/appi.ajp.2013.13060771

[ref14] Dixit A, Marthoenis M, Arafat SMY, Sharma P, Kar SK (2020). Binge watching behavior during COVID 19 pandemic: a cross-sectional, cross-national online survey. Psychiatry Research 113089. 32434095 10.1016/j.psychres.2020.113089 PMC7219409

[ref15] Fusar-Poli P, Bonoldi I, Yung AR, Borgwardt S, Kempton MJ, Valmaggia L, Barale F, Caverzasi E, Mcguire P (2012). Predicting psychosis: meta-analysis of transition outcomes in individuals at high clinical risk. Archives of General Psychiatry 69, 220–229. 22393215 10.1001/archgenpsychiatry.2011.1472

[ref16] Hensel JM, Ellard K, Koltek M, Wilson G, Sareen J (2019). Digital health solutions for indigenous mental well-being. Current Psychiatry Reports 21, 68. 31263971 10.1007/s11920-019-1056-6 PMC6602981

[ref17] Jackson HJ, McGorry PD (2009). The Recognition and Management of Early Psychosis. A Preventive Approach. Cambridge University Press: Cambridge.

[ref18] Joa I, Johannessen JO, Auestad B, Friis S, Mcglashan T, Melle I, Opjordsmoen S, Simonsen E, Vaglum P, Larsen TK (2008). The key to reducing duration of untreated first psychosis: information campaigns. Schizophrenia Bulletin 34, 466–472. 17905788 10.1093/schbul/sbm095 PMC2632428

[ref19] Killackey E (2009). Psychosocial and psychological interventions in early psychosis: essential elements for recovery. Early Intervention in Psychiatry 3 (Suppl. 1), S17–S21. 21352192 10.1111/j.1751-7893.2009.00126.x

[ref20] Kirkbride JB, Croudace T, Brewin J, Donoghue K, Mason P, Glazebrook C, Medley I, Harrison G, Cooper JE, Doody GA, Jones PB (2009). Is the incidence of psychotic disorder in decline? Epidemiological evidence from two decades of research. International Journal of Epidemiology 38, 1255–1264. 18725359 10.1093/ije/dyn168 PMC3307031

[ref21] Krstev H, Carbone S, Harrigan SM, Curry C, Elkins K, McGorry PD (2004). Early intervention in first-episode psychosis – the impact of a community development campaign. Social Psychiatry and Psychiatric Epidemiology 39, 711–719. 15672291 10.1007/s00127-004-0798-5

[ref22] Li J, Yang Z, Qiu H, Wang Y, Jian L, Ji J, Li K (2020). Anxiety and depression among general population in China at the peak of the COVID-19 epidemic. World Psychiatry 19, 249–250. 32394560 10.1002/wps.20758 PMC7214959

[ref23] Linardon J, Cuijpers P, Carlbring P, Messer M, Fuller-Tyszkiewicz M (2019). The efficacy of app-supported smartphone interventions for mental health problems: a meta-analysis of randomized controlled trials. World Psychiatry 18, 325–336. 31496095 10.1002/wps.20673 PMC6732686

[ref24] Marshall M, Lewis S, Lockwood A, Drake R, Jones P, Croudace T (2005). Association between duration of untreated psychosis and outcome in cohorts of first-episode patients: a systematic review. Archives of General Psychiatry 62, 975–983. 16143729 10.1001/archpsyc.62.9.975

[ref25] Mcdermott MM, Newman AB (2020). Preserving clinical trial integrity during the coronavirus pandemic. JAMA 323, 2135–2136. 10.1001/jama.2020.4689 32211830

[ref26] Mcgorry PD, Edwards J, Mihalopoulos C, Harrigan SM, Jackson HJ (1996). EPPIC: an evolving system of early detection and optimal management. Schizophrenia Bulletin 22, 305–326. 8782288 10.1093/schbul/22.2.305

[ref27] Mcgorry PD, Purcell R, Goldstone S, Amminger GP (2011). Age of onset and timing of treatment for mental and substance use disorders: implications for preventive intervention strategies and models of care. Current Opinion in Psychiatry 24, 301–306. 21532481 10.1097/YCO.0b013e3283477a09

[ref28] Nelson B, Yuen H, Wood SJ, Lin A, Spiliotacopoulos D, Bruxer A, Broussard C, Simmons M, Foley DL, Brewer WJ, Francey SM, Amminger PG, Thompson A, McGorry PD, Yung AR (2013). Long-term follow-up of a group at ultra high risk (‘prodromal’) for psychosis: the pace 400 study. JAMA Psychiatry 70, 793–802. 23739772 10.1001/jamapsychiatry.2013.1270

[ref29] O’Callaghan E, Turner N, Renwick L, Jackson D, Sutton M, Foley SD, Mcwilliams S, Behan C, Fetherstone A, Kinsella A (2010). First episode psychosis and the trail to secondary care: help-seeking and health-system delays. Social Psychiatry and Psychiatric Epidemiology 45, 381–391. 19578801 10.1007/s00127-009-0081-x

[ref30] O’Donoghue B, Lyne J, Kinsella A, Turner N, O’Callaghan E, Clarke M (2014). Detection and characteristics of individuals with a very long duration of untreated psychosis in an early intervention for psychosis service. Early Intervention in Psychiatry 8, 332–339. 23786447 10.1111/eip.12063

[ref31] O’Donoghue B, Lyne J, Renwick L, Madigan K, Kinsella A, Clarke M, Turner N, O’Callaghan E (2012). A descriptive study of ‘non-cases’ and referral rates to an early intervention for psychosis service. Early Intervention in Psychiatry 6, 276–282. 22240056 10.1111/j.1751-7893.2011.00328.x

[ref32] O’Donoghue B, Rudhran V, Kumar S, Bowtell M, Polari A, Mackinnon A, Mcgorry P, Nelson B (2018). Screening for the ultra-high risk state in a youth mental health service. Schizophrenia Research 202, 401–403. 30539772 10.1016/j.schres.2018.06.045

[ref33] Power P (2019). Early intervention services for psychosis in Ireland: are we there yet? Irish Journal of Psychological Medicine 36, 243–248. 31747987 10.1017/ipm.2019.44

[ref34] Prem K, Liu Y, Russell TW, Kucharski AJ, Eggo RM, Davies N, Jit M, Klepac P (2020). The effect of control strategies to reduce social mixing on outcomes of the COVID-19 epidemic in Wuhan, China: a modelling study. Lancet Public Health 5, e261–e270. 32220655 10.1016/S2468-2667(20)30073-6 PMC7158905

[ref35] Robinson J, Hetrick SE, Martin C (2011). Preventing suicide in young people: systematic review. Australian and New Zealand Journal of Psychiatry 45, 3–26. 21174502 10.3109/00048674.2010.511147

[ref36] Ross R, Neeland IJ, Yamashita S, Shai I, Seidell J, Magni P, Santos RD, Arsenault B, Cuevas A, Hu FB, Griffin BA, Zambon A, Barter P, Fruchart JC, Eckel RH, Matsuzawa Y, Despres JP (2020). Waist circumference as a vital sign in clinical practice: a Consensus Statement from the IAS and ICCR Working Group on Visceral Obesity. Nature Reviews Endocrinology 16, 177–189. 10.1038/s41574-019-0310-7 PMC7027970 32020062

[ref37] Santesteban-Echarri O, Paino M, Rice S, Gonzalez-Blanch C, Mcgorry P, Gleeson J, Alvarez-Jimenez M (2017). Predictors of functional recovery in first-episode psychosis: a systematic review and meta-analysis of longitudinal studies. Clinical Psychology Review 58, 59–75. 29042139 10.1016/j.cpr.2017.09.007

[ref38] Schimmelmann BG, Huber CG, Lambert M, Cotton S, Mcgorry PD, Conus P (2008). Impact of duration of untreated psychosis on pre-treatment, baseline, and outcome characteristics in an epidemiological first-episode psychosis cohort. Journal of Psychiatric Research 42, 982–990. 18199456 10.1016/j.jpsychires.2007.12.001

[ref39] Shah JL, Malla AK (2015). Much ado about much: stress, dynamic biomarkers and HPA axis dysregulation along the trajectory to psychosis. Schizophrenia Research 162, 253–260. 25620122 10.1016/j.schres.2015.01.010

[ref40] Sutton M, O’Keeffe D, Frawley T, Madigan K, Fanning F, Lawlor E, Roche E, Kelly A, Turner N, Horenstein A, O’Callaghan E, Clarke M (2018). Feasibility of a psychosis information intervention to improve mental health literacy for professional groups in contact with young people. Early Intervention in Psychiatry 12, 234–239. 28102617 10.1111/eip.12410

[ref41] Too LS, Spittal MJ, Bugeja L, Reifels L, Butterworth P, Pirkis J (2019). The association between mental disorders and suicide: a systematic review and meta-analysis of record linkage studies. Journal of Affective Disorders 259, 302–313. 31450139 10.1016/j.jad.2019.08.054

[ref42] Torous J, Wykes T (2020). Opportunities from the coronavirus disease 2019 pandemic for transforming psychiatric care with Telehealth. JAMA Psychiatry. Published online ahead of print. doi:10.1001/jamapsychiatry.2020.1640. 32391857

[ref43] Turner N, Foley SR, Kinsella A, O’Callaghan E, Clarke M (2014). Putting television’s portrayal of schizophrenia into reverse: an evaluation of the impact on public opinion. Early Intervention in Psychiatry 8, 366–374. 23773257 10.1111/eip.12056

[ref44] Yung AR, Nelson B (2013). The ultra-high risk concept – a review. The Canadian Journal of Psychiatry 58, 5–12. 23327750 10.1177/070674371305800103

